# Tumor location as an indicator of survival in T1 resectable pancreatic ductal adenocarcinoma: a propensity score-matched analysis

**DOI:** 10.1186/s12876-019-0975-3

**Published:** 2019-04-24

**Authors:** Zibo Meng, Mingsi Cao, Yushun Zhang, Zhiqiang Liu, Shihong Wu, Heshui Wu

**Affiliations:** 1 Department of Pancreatic Surgery, Union Hospital, Tongji Medical College, Huazhong University of Science and Technology, Wuhan, 430022 China; 2 Institute of Cardiology, Union Hospital, Tongji Medical College, Huazhong University of Science and Technology, Wuhan, 430022 China

**Keywords:** Pancreatic ductal adenocarcinoma, Tumor location, Survival, Propensity score-matched analysis

## Abstract

**Background:**

The latest 8th edition of the AJCC staging system emphasizes the importance of tumor size however, the clinical significance of the combination of tumor location with tumor size remains unknown.

**Methods:**

We conducted this study to investigate the prognostic role of tumor location in T1 resectable pancreatic ductal adenocarcinoma (PDAC). Resectable PDAC patients from Surveillance, Epidemiology, and End Results (SEER) database (2004–2014) were selected for the propensity score matching analysis. We used matched cohort to analyze the relationship between clinicopathologic features and survival of patients.

**Result:**

Eight thousand, four hundred nine patients were included in the propensity score matching analysis and 4571 patients were selected for final analysis. In T1 patients, the patients with pancreatic head tumor had worse prognosis compared to the patients with body/tail tumors. Multivariate analysis result showed that pancreatic body/tail location was an independent indicator for better chances of survival in T1 PDAC patients (hazard ratio, 0.69; 95%CI, 0.52–0.93; *P* = 0.01). The modified staging system was more efficient than the AJCC 8th staging system.

**Conclusion:**

Modified staging system exhibited a good assessment of the survival rate. The tumor location is a good prognostic indicator for T1 resectable PDAC patients. Modification of T1 subgroup according to tumor location exhibited favorable survival prediction effects.

## Background

Pancreatic ductal adenocarcinoma (PDAC) is a highly lethal disease. It has become the fourth-leading cause of cancer-related deaths and is projected to become the second leading cause of cancer-related deaths in the United States by 2030 [1, 2]. It is refractory to most treatment and exhibits a general 5-year survival rate of 8% [2, 3]. For now, surgical resection is the only potential treatment for PDAC patients [4]. Hence, it is important to assess the extent of tumor progression to determine the suitable surgical procedure. The American Joint Committee on Cancer (AJCC) staging system is the most widely used indicator in malignancies prognosis predictions. In the latest 8th edition of the AJCC staging system, the importance of tumor size for patients’ prognosis is further emphasized in T-staging systems. Resectable PDACs are more likely to have a smaller tumor volume than extensively metastatic tumors, and the selection of appropriate surgical treatment for resectable PDAC, especially T1 tumors, is very urgent.

Different tumor locations have different infiltration periods in blood vessels and the surrounding organs. Due to their anatomical location, pancreatic tumors located in the head and body/tail require very different surgical methods. Several studies suggested that the location of the tumor may have prognostic value for PDAC, but no consensus has been reached [5–7]. In addition, the 8th edition of the AJCC staging system for PDAC does not consider the impact of the location of the tumor. The aim of our study is to find out the influence of the tumor location on prognosis, and propose modifications for the 8th AJCC T-staging system in PDAC.

## Methods

### Patients and data collection

The Surveillance, Epidemiology, and End Results (SEER) database (2004–2014) of the US National Cancer Institute was utilized for this research. We used SEER*Stat software (Version 8.3.4) to retrieve the patient’s data. Patients with PDAC were identified using the topography codes (C25.0-C25.4 and C25.7-C25.9) and histology codes (8500/3 and 8140/3) of the International Classification of Diseases for Oncology, third edition (ICD-O-3). Other variants of pancreatic malignancies were excluded. Patients with undefined tumor size or unclear tumor location (for example overlapping lesion of pancreas, pancreatic duct, pancreatic neck, and other unspecific locations), who were younger than 18 years old, and did not have surgeries performed on them, were excluded in our study. We collected demographic data, including age, race, and gender. Tumor information, including tumor location, size, grade, AJCC 7th TNM stages, lymph nodes information, and operation methods were retrieved. We extracted survival information such as number of months survived, causes of deaths, and vital statuses. We transformed the AJCC 7th stage into AJCC 8th stage according to the definition of these two systems [8]. Since our data was from SEER public database, no specific patient’s information was recorded so ethical consent was not necessary.

### Statistical analysis

Statistical data were analyzed using SPSS software version 20.0 (SPSS Inc., Chicago, IL, USA) and MedCalc Statistical Software version 15.2.2 (MedCalc Software bvba, Ostend, Belgium; http://www.medcalc.org; 2015). Continuous variables were shown as means and standard deviations, while categorical variables were exhibited as frequencies and percentages. The survival month was calculated according to the time from diagnosis to the data of death or the last follow-up. In order to simulate the randomized controlled trials and reduce the effect of selection bias, a 1-to-3 propensity score matching method was conducted using the nearest-neighbor method with a stringent caliper of 0.05 [9]. We used Kaplan-Meier curves with log-rank test to perform the survival analysis. To determine the prognostic factors of resectable PDAC, we used Cox proportional-hazards model with backwards-stepwise selection [10]. The results of multivariate regression analysis and univariate regression analysis were demonstrated as hazard ratios (HR) and 95% confidence intervals (CI). We also used receiver operating characteristic (ROC) curve analysis to compare the discriminatory ability of the 2 staging systems [11]. All tests were 2-sided and a *p* < 0.05 was considered statistically significant.

## Results

### Clinicopathologic characteristics

**Fig. 1:**
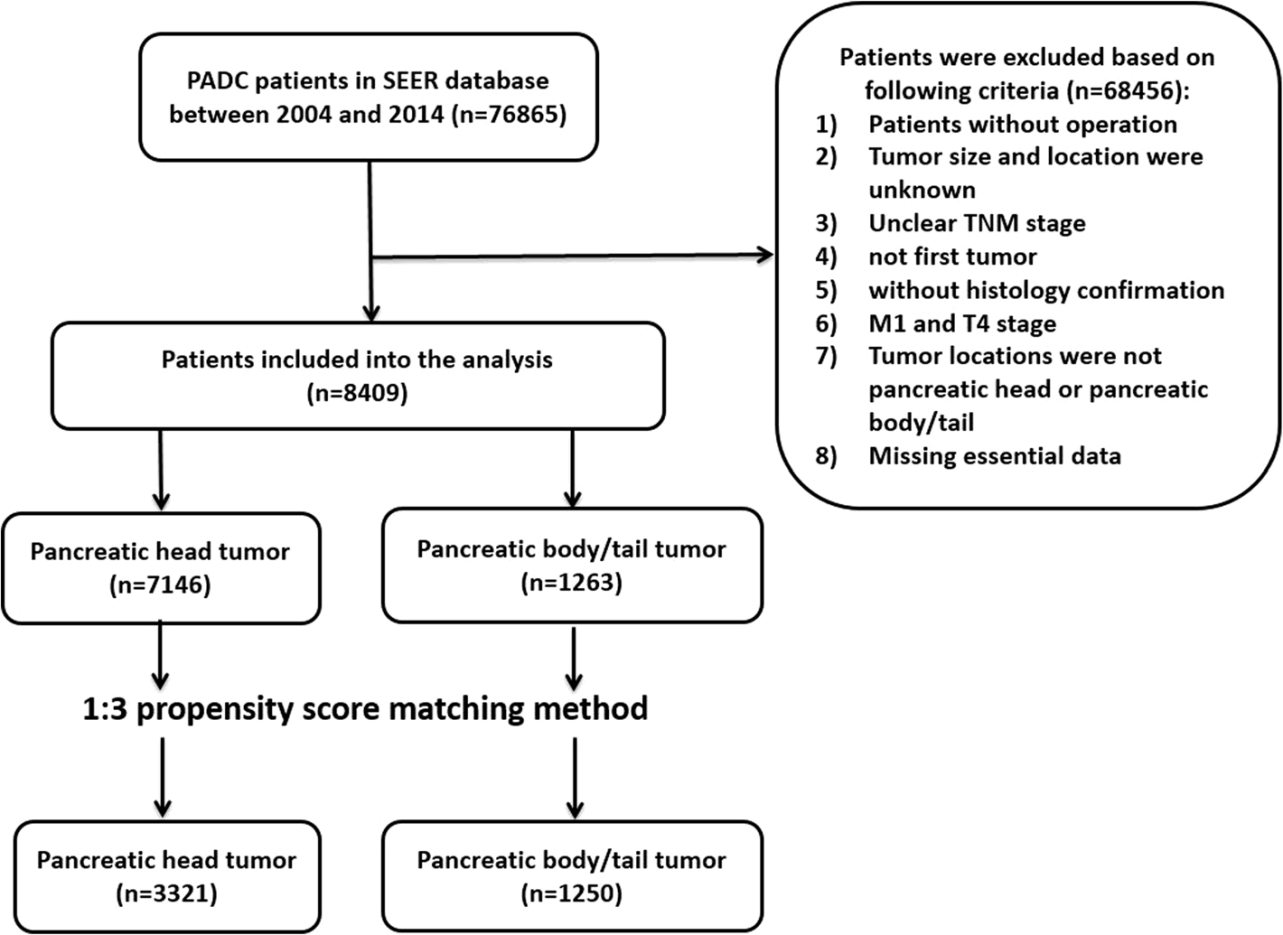
Flowchart of this study

As shown in Fig. 1, a total of 8409 PDAC patients were brought into analysis under the above inclusion and exclusion criteria. Among them, 7146 patients had pancreatic head PDACs and 1263 patients had pancreatic body/tail tumors. The original group showed a significant difference between pancreatic head group and pancreatic body/tail group in baseline characteristics (Table 1). Then, we used this cohort to apply the 1-to-3 propensity score matching based on the year of diagnosis, race, gender, marital status, T stage, N stage, and pathology grade. The matched cohort (*n* = 4571) contained 3321 patients with pancreatic head tumor and 1250 patients with pancreatic body/tail tumors. The baseline variables were matched very well between two groups except T stage so we conducted subgroup analysis based on T stage.

**Table 1:** Clinical features between pancreatic head PAAD and pancreatic body/tail PAAD in original and matched cohorts

Original cohort (*n* = 8409)				Matched cohort (*n* = 4571)			
Characteristics	Head	Body/Tail	*P*	Characteristics	Head	Body/Tail	*P*
Age at diagnosis	65.3 ± 10.4	66.4 ± 10.8	< 0.001	Age at diagnosis	66.2 ± 10.3	66.4 ± 10.8	0.540
Year of diagnosis			< 0.001	Year of diagnosis			0.499
2004–2008	2813	431		2004–2008	1170	427	
2009–2014	4333	832		2009–2014	2151	823	
Race			< 0.001	Race			0.934
White	5900	982		White	2615	977	0.032
Yellow	478	114		Yellow	281	110	
Black	706	158		Black	397	154	
Others	62	9		Others	28	9	
Gender				Gender			
Female	3542	662	0.062	Female	1706	651	0.669
Male	3604	601		Male	1615	599	
Marital status				Marital status			
Married	4516	805	0.319	Married	2095	795	0.891
Single	840	128		Single	367	127	
Divorced	741	125		Divorced	320	125	
Widowed	849	163		Widowed	437	161	
Unknown	200	42		Unknown	102	42	
T stage				T stage			
T1	1281	177	< 0.001	T1	492	177	< 0.001
T2	4497	559		T2	1680	559	
T3	1368	527		T3	1149	514	
N stage			< 0.001	N stage			0.087
N0	2233	606		N0	1469	595	
N1	3037	476		N1	1307	474	
N2	1876	181		N2	545	181	
Pathology grade			0.171	Pathology grade			0.947
Grade 1	654	123		Grade 1	301	121	
Grade 2	3488	649		Grade 2	1733	638	
Grade 3	2523	403		Grade 3	1063	403	
Grade 4	59	14		Grade 4	35	14	
Unknown	422	74		Unknown	189	74	

### The relationship between tumor location and CSS in matched cohort

Of the 4571 PDAC patients in matched cohort, 3321 (72.7%) patients had pancreatic head whereas 1250 (27.3%) patients had pancreatic head/tail tumors. In T1 PDAC patients, 73.5% (492/669) tumors originated in the pancreatic head. 25.0% (559/2239) T2 patients had pancreatic head PDAC and 69.1% (1149/1663) T3 tumors occurred in the pancreatic head.

**Fig. 2:**
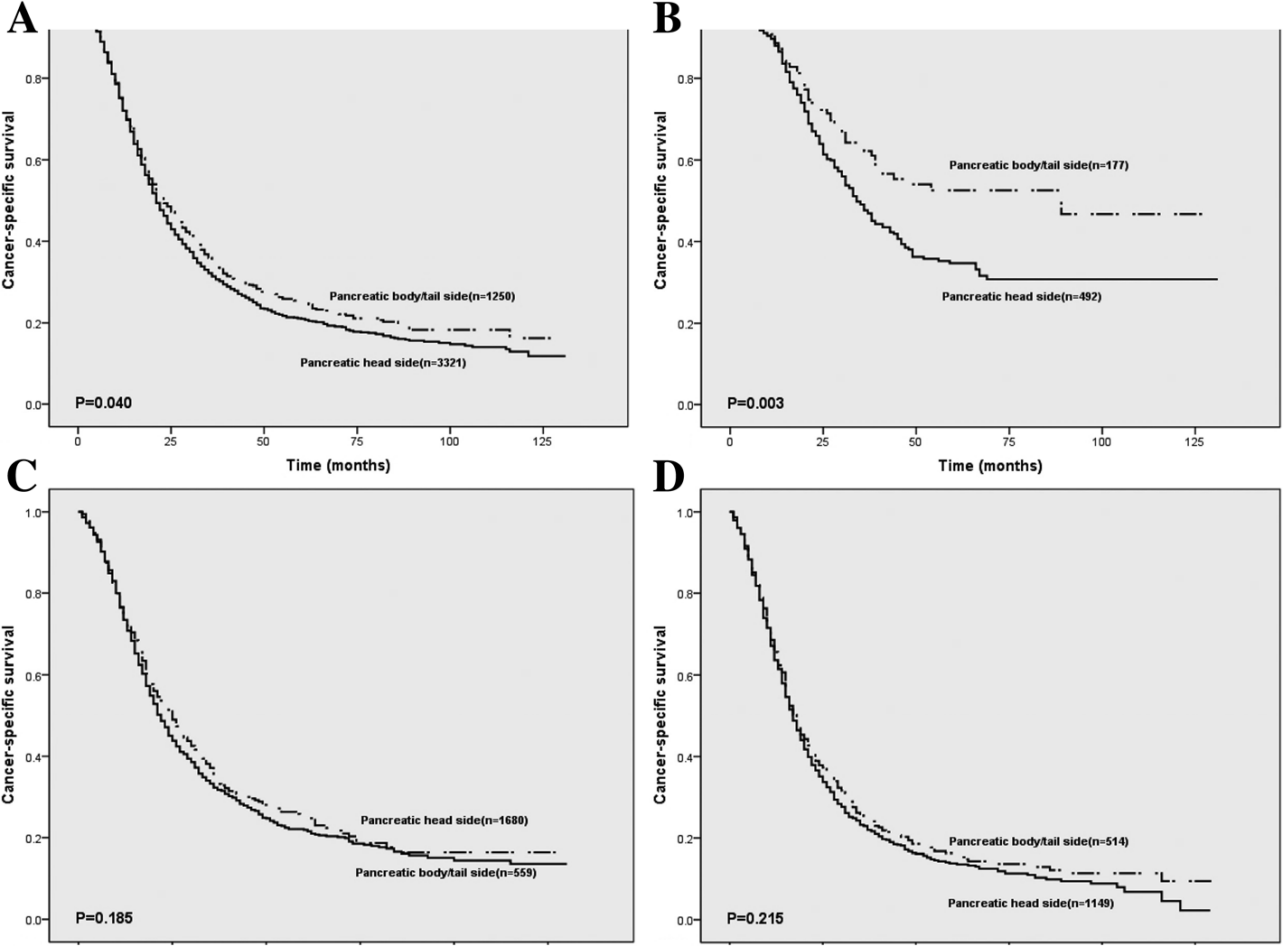
Prognostic effect of tumor location in resectable PDAC according to T stage. **a**. T1-T3 stage; **b**. T1 stage; **c**. T2 stage; **d**. T3 stage

As is shown in Fig. 2, we compared CSS between the head and body/tail subgroups. The survival rate of pancreatic head PDAC patients in T1 (*p* = 0.003) was significantly poorer. In T1 group, 1, 3 and 5-years survival rates were 88.0, 47.6 and 34.6%, respectively. For pancreatic head tumors (T1 h); 1, 3 and 5-years survival rates were 88.7, 62.2 and 52.6% for pancreatic body/tail tumors (T1b/t), respectively. Interestingly, there was no significant correlation between tumor location and survival in patients with PDAC in T2 (*p* = 0.185) and T3 (*p* = 0.215) patients. These results showed that tumor location is associated with survival in patients with T1 PDAC.

### Correspondence between tumor location and histopathology characters in T1 stage

Using the matched group, we explored the histopathological evidence because of the poor survival of pancreatic head PDAC patients. Positive lymph nodes and tumor size information were further used to make comparisons between the head and body/tail subgroups. In the matched two groups, patients had similar lymph nodes positive rate (*p* = 0.053) and positive lymph nodes (*p* = 0.147) in T1 stage. Although we limited the size of the tumor to no more than 2 cm, the average tumor size was larger in the pancreatic head group (1.66 cm vs. 1.48 cm, *P* < 0.001).

Because we used a well matched cohort, the year of diagnosis, race, age, gender, marital status, N stage and pathology grades showed no significant difference between two groups.

### Prognostic factors for patients with early T1 PDAC

We performed univariate and multivariate analysis to determine the prognostic indicators for the early T-stage PDAC. 9 potential factors were selected: the year of diagnosis, size, age, gender, N stage, tumor location, pathology grade, race and marital status (Table 2). Among these, the year of diagnosis, N stage, and tumor location was statistically significant.

**Table 2:** Prognostic factors for T1 PAAD patients. HR: hazard ratio. CI: confidence interval

	Univariate analysis							Multivariate analysis					
					95%CI							95%CI	
	Standard error	Wald chi-square	P	HR	Down	Up		Standard error	Wald chi-square	*P*	HR	Down	Up
Year of diagnosis							Year of diagnosis						
2004–2008	Reference						2004–2008	Reference					
2009–2014	0.12	18.96	< 0.001	0.60	0.47	0.75	2009–2014	0.12	18.62	< 0.001	0.60	0.47	0.76
Race							Race						
White	Reference						White						
Yellow	0.22	0.99	0.32	0.81	0.53	1.23	Yellow						
Black	0.18	0.07	0.79	1.05	0.73	1.51	Black						
Others	0.71	0.43	0.51	0.63	0.16	2.53	Others						
Age	0.01	0.11	0.74	1.00	0.99	1.01	Age						
Gender							Gender						
Male	Reference						Male						
Female	0.12	1.66	0.20	0.86	0.68	1.08	Female						
Marital status							Marital status						
Married	Reference						Married						
Single	0.23	2.50	0.11	0.70	0.45	1.09	Single						
Divorced	0.21	0.11	0.74	0.93	0.62	1.41	Divorced						
Widowed	0.16	1.22	0.27	1.19	0.87	1.62	Widowed						
Unknown	0.34	0.14	0.71	0.88	0.45	1.72	Unknown						
Size	0.02	8.89	0.003	1.05	1.02	1.08	Size						
N stage							N stage						
N0	Reference						N0	Reference					
N1	0.13	26.95	< 0.001	1.92	1.50	2.45	N1	0.13	17.52	< 0.001	1.71	1.33	2.19
N2	0.23	12.17	< 0.001	2.22	1.42	3.47	N2	0.23	11.28	0.001	2.17	1.38	3.40
Site							Site						
Head	Reference						Head	Reference					
Body/Tail	0.15	8.55	0.003	0.65	0.49	0.87	Body/Tail	0.15	6.18	0.01	0.69	0.52	0.93
Pathology grade							Pathology grade						
Grade 1	Reference						Grade 1	Reference					
Grade 2	0.17	1.96	0.16	1.28	0.91	1.79	Grade 2	0.18	1.17	0.28	1.21	0.86	1.70
Grade 3	0.19	9.06	0.003	1.79	1.23	2.62	Grade 3	0.20	7.19	0.01	1.70	1.15	2.50
Grade 4	0.60	0.85	0.36	1.73	0.54	5.60	Grade 4	0.60	0.72	0.40	1.66	0.51	5.37
Unknown	0.28	0.42	0.52	1.20	0.69	2.09	Unknown	0.28	0.55	0.46	1.23	0.71	2.15

We included factors whose *p* < 0.1 in univariate analysis and parameter with important clinical significance (pathology grade) into multivariate analysis. Among them, tumor location, year of diagnosis, and N stage were independent prognostic indicators for T1 PDAC patients.

### Proposed modification of 8th edition AJCC T-staging system based on the tumor location

**Fig. 3:**
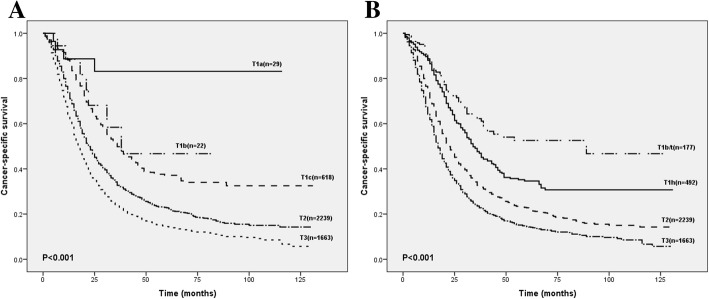
**a**. Comparison of survival according to T stage with subclassification of tumors by the 8th edition of the AJCC T-staging system. **b**. Comparison of survival according to T stage with subclassification of tumors by the tumor location

Through analyzing the survival rate and median/mean CSS of patients in each sub-stage from SEER cohort, we found some insufficiencies in the current 8th edition AJCC T-staging system. As shown in Table 3, the average CSS rate of T1c stage was higher than that of T1b stage (61.4 vs. 51.4). The survival curve showed that the short-term survival of PDAC were not clearly distinguished among each group, especially T1 (Fig. 3a). Existing 8th AJCC T-staging system had some shortages that needed to be improved.

**Table 3:** Patients’ survival rates in different T-staging systems

AJCC 8th T stage							Modified 8th T stage						
		Survival rates, %			Survival (month)				Survival rates, %			Survival (month)	
	*n*	1 year	3 years	5 years	mean	median		n	1 year	3 years	5 years	mean	median
T1a	29	88.7	83.1	83.1	98.6	–	T1b/t	177	88.7	62.2	52.6	76.6	89
T1b	22	88.5	58.4	46.7	51.4	38	T1 h	492	88.0	47.6	34.6	58.8	34
T1c	618	88.2	49.8	37.2	61.4	36							
T2	2239	73.7	32.7	22.8	41.0	22	T2	2239	73.7	32.7	22.8	41.0	22
T3	1663	64.2	23.5	14.5	31.1	17	T3	1663	64.2	23.5	14.5	31.1	17

We subdivided the T1 PDAC according to the tumor location. The modified T classification effectively distinguished patients’ prognosis (Fig. 3b). Compared with the current AJCC 8th edition T-staging system, the survival rate was significantly different from each period in modified systems and average CSS varied evidently. The modified T-staging system showed a better diagnostic effect. The results were 0.535 (95% CI, 0.496–0.573) for 8th T-staging system and 0.559 (95% CI, 0.520–0.597) for modified T-staging system.

### Effect of modified T-stage on the 8th edition of AJCC system

**Fig. 4:**
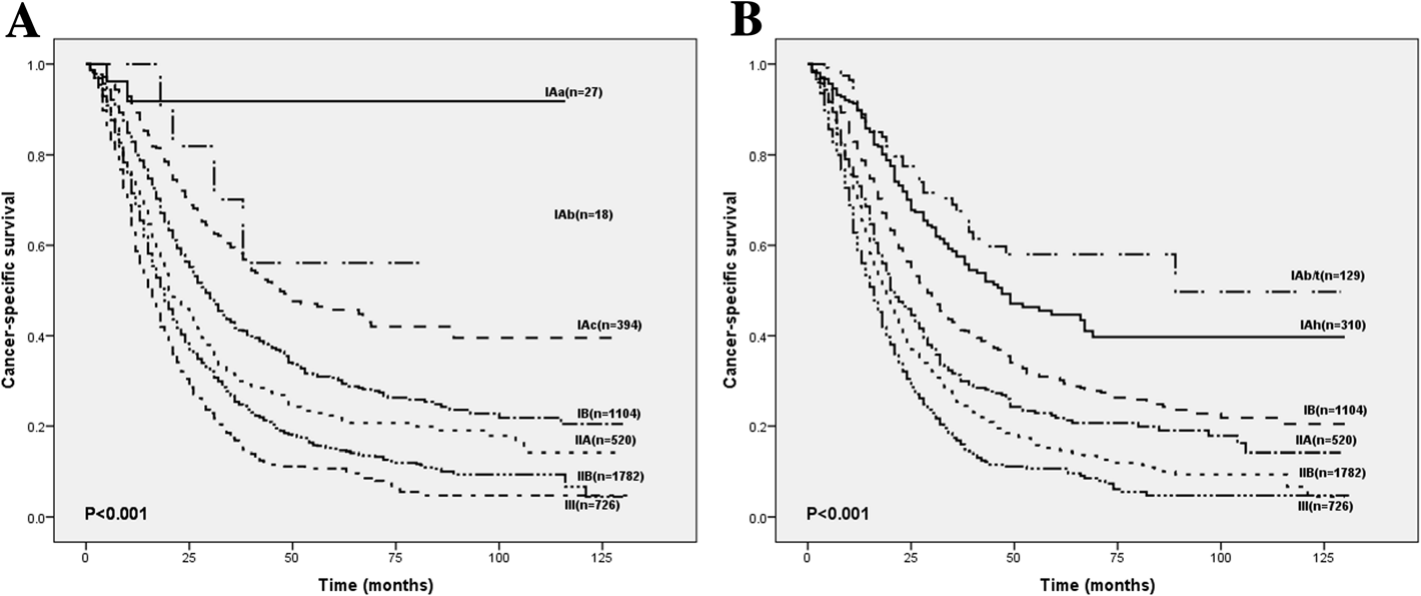
**a**. Comparison of survival according to conventional TNM classification with subclassification of early T-stage tumors by the 8th edition of the AJCC T-staging system. **b**. Comparison of survival according to conventional TNM classification with subclassification of early T-stage tumors by the modified T-staging system

The modification of T1 stage mainly affected the stage IA (T1N0M0). We subdivided stage IA into T1b/tN0M0 (IAb/t) and T1 hN0M0 (IAh). As shown in Fig. 4, compared with the survival curves using the current AJCC system, the modified staging system effectively discriminated the stage IA, especially the short-term survival rate. Within AJCC 8th edition system, the 1-year CSS rate in stage IA didn’t produce significant differences in survival with size changes. Group IAc had a better average CSS compared with IAb group (69.7 vs. 59.1). However, the 1-year, 3-year and 5-year survival rate, respectively, declined gradually with the modified stages, respectively. It can be seen that the modified classification successfully stratified the patients’ prognosis. AUC analysis result for the 8th staging system was 0.570(95% CI, 0.532–0.608) and 0.611(95% CI, 0.573–0.648) for the modified staging system, and the difference between two curves was 0.041(95% CI, 0.018–0.063, *p* < 0.001) which meant that the prognostic effect of the modified staging system is better than that of the 8th edition AJCC staging system.

## Discussion

In our study, we investigated the prognostic value of tumor location for T1 resectable PDAC patients. The pancreatic head location was associated with the bigger size and had worse survival rates compared with the pancreatic body/tail location. To the best of our knowledge, it is the first indication that the pancreatic head location was an indicator for a bad outcome in T1 resectable PDAC.

The newly-introduced 8th edition of the AJCC staging system highlights the importance of tumor size for T staging systems, and also. In addition, T1 (≤2 cm) patients are a considerable part of the patients who can undergo surgical treatment which is why it is crucial to identify the prognostic factors of these patients. As displayed in Table 2, the year of diagnosis and N stage were well-known prognostic factors. Locations of tumors, which can be evaluated before surgery, have a direct effect on the patients’ prognosis.

For now, the prognostic significance of tumor location in pancreatic cancer is still controversial. Several researches reported that tumors located at the body and tail of the pancreas had higher mortality risks. An explanation for these results is that the timing of diagnosis or lead-time bias may result in the difference between pancreatic head and pancreatic body/tail tumors. Patients with pancreatic head tumors gained additional period from earlier diagnosis, rather than tumor biology or intervention differences [12]. Ling et al. found out that patients with pancreatic body/tail tumors had a higher rate of overall-survival and tumor-free survival [13]. Another study reported that local-stage pancreatic body/tail cancer patients had better survival rates compared with local-stage pancreatic head cancer patients [14]. Pancreatic body and tail tumors, due to the late symptoms, give us a subjective impression that it tends to be more advanced, larger, and worse prognoses for patients [15–17]. Ruess et al. showed that although the tumor sizes were larger, the prognosis of resectable pancreatic body and tail PDAC patients were similar to that of pancreatic head PDAC [18]. Our result exhibited that pancreatic head PDAC had a bigger tumor size, and in the T1 stage, pancreatic body/tail PDAC patients had better survival rates compared to the pancreatic head group. Several reasons may be a factor of this phenomenon. Firstly, the tumor site is the primary determinant of the surgical approach and different surgical methods have an impact on the patient’s gastrointestinal function which has distinct complications that determined patients’ outcome-especially on short-term survival [19, 20]. Radical antegrade modular pancreatosplenectomy, reported in 2003, may get higher margin-free resection rates which improved the prognosis after distal pancreatic tumor resection [21, 22]. Secondly, according to the Japan Pancreas Society, it is stated that in the nomenclature of peripancreatic lymph nodes, the pancreatic head has a more complex lymphatic drainage system compared with the distal pancreas, and the extent of lymphadenectomy during pancreatectomy for pancreatic head PDAC is still disputed [23, 24]. Several studies also showed that pancreatic body/tail tumors have less frequent nodal involvement, which could explain the better outcome of these patients [25, 26]. In our results after the propensity score matching, two groups had similar number of positive lymph nodes and lymph nodes positive rate. The survival between groups were still different, which showed that the impact of location cannot be ignored. The pancreatic head is adjacent to many important organs and blood vessels. Compared with the resection of pancreatic head tumors which have a greater impact on important structures, multivisceral resection of the distal pancreatic tumor is safer and more feasible [27–29]. What’s more, pancreatic head PDAC can lead to the malignant biliary obstruction and complications after preoperative biliary drainage which cause the delay or even omission of surgery [30]. Hyoun et al. reported that tumors in proximal locations, although smaller in size, were more dedifferentiated than distal tumors which resulted in poor prognosis [26]. Ling et al. reported that pancreatic body/tail PDAC had remarkably lower expressions of miR-501-3p compared with the pancreatic head one. The in vivo and in vitro experiments proved that miR-501-3p could enhance the invasiveness of PDAC cells and resulted in tumor recurrence [13]. Another Study also showed that pancreatic head and pancreatic body/tail PDAC had distinct genetic and molecular features [31].We also improved the subcategories of T1 staging according to the tumor locations. The modified T-staging system exhibited good patient survival stratification for it had better prognosis-discrimination effect than the 8th AJCC staging system. The significance of AJCC staging system is to accurately stratify the patients’ prognosis. Therefore, we proposed the subgroups of T1 according to the tumor locations to promote the precision of AJCC staging system. Compared with the current AJCC 8th edition system, our modified T-staging and corresponding TNM staging system effectively separated the survival curves between stages, especially for the differentiation of short-term survival of patients. There are some advantages of this modified staging system. From a clinical point of view, the determination of tumor location is relatively easy and has more operability in clinical practice. Moreover, the majority of patients are diagnosed at an advanced stage [32, 33], and patients with < 1 cm tumors account for a very small proportion of total patients which can also be observed in our cohort. Hence, the size-based sub-stage of T1 may not be practical in clinical work.

There are also some limitations of this study: 1) only selected SEER cohort; 2) lacked a validation group; 3) only selected resectable PDAC which occurred in the head and the body/tail of the pancreas (excluding patients with PDAC in the pancreatic neck, overlapping lesion of pancreas and other unclear locations); 4) lacked small-sized tumor patients; 5) Multi-center prospective study needed to be applied for further studies; 6) The SEER database was only able to provide basic clinical information hence our research lacked the exploration of further biological mechanisms.

## Conclusions

Tumor location is a predictor of resectable ≤2 cm PDAC. Modification of T1 sub stage according to tumor location is accurate and effective in prognostic prediction of PDAC.

## Abbreviations

95%CI: 95% confidence intervals
AJCC: the American Joint Committee on Cancer
C-index: concordance index
CSS: cancer-specific-survival
HR: hazard ratios
PDAC: pancreatic ductal adenocarcinoma
SEER database: Surveillance, Epidemiology, and End Results database
T1 h: T1-stage pancreatic-head-side tumors
T1b/t: T1-stage pancreatic-body/tail-side tumors
